# The Association of Circulating CD14++CD16+ Monocytes, Natural Killer Cells and Regulatory T Cells Subpopulations With Phenotypes of Cardiovascular Disease in a Cohort of Peritoneal Dialysis Patients

**DOI:** 10.3389/fmed.2021.724316

**Published:** 2021-10-20

**Authors:** Anila Duni, Georgios Vartholomatos, Olga Balafa, Margarita Ikonomou, Paraskevi Tseke, Lampros Lakkas, Karolos Paulos Rapsomanikis, Athanasios Kitsos, Ioanna Theodorou, Charalambos Pappas, Katerina K. Naka, Michael Mitsis, Evangelia Dounousi

**Affiliations:** ^1^Department of Nephrology, University Hospital of Ioannina, Ioannina, Greece; ^2^Laboratory of Haematology - Unit of Molecular Biology, University Hospital of Ioannina, Ioannina, Greece; ^3^Renal Unit, General Hospital Alexandra, Athens, Greece; ^4^Second Department of Cardiology and Michaelidion Cardiac Center, Medical School University of Ioannina, Ioannina, Greece; ^5^Department of Surgery, University Hospital of Ioannina, Ioannina, Greece; ^6^Department of Surgery, School of Medicine, University of Ioannina, Ioannina, Greece; ^7^Department of Nephrology, School of Medicine, University of Ioannina, Ioannina, Greece

**Keywords:** CD14++CD16+ monocytes, natural killer cells, CD4+CD25+ regulatory T cells, coronary artery disease, overhydration, fast transporters

## Abstract

The altered expression of immune cells including monocyte subsets, natural killer (NK) cells and CD4+CD25+ regulatory T cells (Tregs) in end-stage kidney disease, affect the modulation of inflammation and immunity with significant clinical implications. The aim of this study was to investigate the profile of specific immune cells subpopulations and their correlations with phenotypes of established cardiovascular disease (CVD), including coronary artery disease (CAD) and heart failure (HF) in peritoneal dialysis (PD) patients.

**Materials and Methods:** 29 stable PD patients and 13 healthy volunteers were enrolled. Demographic, laboratory, bioimpedance measurements, lung ultrasound and echocardiography data were collected. The peripheral blood immune cell subsets analysis was performed using flow cytometry.

**Results:** PD patients compared to normal controls had lower total lymphocytes (22.3 ± 6.28 vs. 31.3 ± 5.54%, *p* = <0.001) and B-lymphocytes (6.39 ± 3.75 vs. 9.72 ± 3.63%, *p* = 0.01) as well as higher CD14++CD16+ monocytes numbers (9.28 ± 6.36 vs. 4.75 ± 2.75%, *p* = 0.0002). PD patients with prevalent CAD had NK cells levels elevated above median values (85.7 vs. 40.9%, *p* = 0.04) and lower B cells counts (3.85 ± 2.46 vs. 7.2 ± 3.77%, *p* = 0.03). Patients with increased NK cells (>15.4%) had 3.8 times higher risk of CAD comparing with patients with lower NK cell levels (95% CI, 1.86 – 77.87; *p* = 0.034). B cells were inversely associated with the presence of CAD (increase of B-lymphocyte by 1% was associated with 30% less risk for presence of CAD (95% CI, −0.71 – 0.01; *p* = 0.05). Overhydrated patients had lower lymphocytes counts (18.3 ± 4.29% vs. 24.7 ± 6.18%, *p* = 0.006) and increased NK cells [20.5% (14.3, 23.6) vs. 13.21% (6.23, 19.2), *p* = 0.04)]. In multiple logistic regression analysis the CRP (OR 1.43; 95% CI, 1.00 – 2.05; *p* = 0.04)] and lymphocytes counts (OR 0.79; 95% CI, 0.63–0.99; *p* = 0.04)] were associated with the presence of lung comets. Patients with higher NK cells (>15.4%, *n* = 15) were more likely to be rapid transporters (D/P creatinine 0.76 ± 0.1 vs. 0.69 ± 0.08, *p* = 0.04). Patients displaying higher Tregs (>1.79%) were older (70.8 ± 10.7 years vs. 57.7 ± 14.7years, *p* = 0.011) and had higher nPCR (0.83 ± 0.14 vs. 0.91 ± 0.17, *p* = 0.09).

**Conclusion:** Future research is required to evaluate the role of immune cells subsets as potential tools to identify patients at the highest risk for complications and guide interventions.

## Introduction

The chronic inflammatory state is considered a hallmark of end-stage kidney disease (ESKD) and is considered to play a pivotal part in the pathogenesis and progression of the compound phenotypes of cardiovascular disease in chronic kidney disease (CKD), including accelerated atherosclerosis, left ventricular hypertrophy (LVH) and heart failure (1).

The complex derangement of the innate and acquired arms of the immune system in patients with CKD includes a vast array of pathogenic mechanisms and effectors. It has been suggested that the altered expression of the immune cells including monocyte subsets, natural killer (NK) cells as well as CD4+CD25+ regulatory T cells (Tregs) affects the modulation of inflammation and immunity with significant clinical implications (2). The three phenotypically and functionally distinct human monocyte subsets are specified by the expression of CD14 and CD16 surface antigens and include CD14++CD16– (classical), CD14++CD16+ (intermediate) and CD14+CD16++ (non-classical) monocytes (3). The pro-inflammatory CD14++CD16+ intermediate monocytes are characterized by upregulated chemokine receptors relevant to atherosclerosis, a high capacity for oxidized low-density lipoprotein (LDL) uptake as well as proangiogenic properties (3, 4). NK cells, apart from being essential players in innate immunity pathways, are currently considered to perform important functions that bridge the innate and acquired arms of the immune system, thus arranging adaptive immune responses and immunoregulation (5). Although their direct role in atherogenesis has been delineated, data regarding their role in heart failure are limited (6). CD4+CD25+FOXP3+ regulatory T cells (Tregs) are a specific subpopulation of T cells, comprising 5–10% of all peripheral CD4+ T cells. They hold a key position in the regulation of the intertwining pathways of immune homeostasis and tolerance with available evidence indicating a potential protective role against cardiovascular disease (7).

The pathophysiology of the chronic inflammatory state of ESKD in Peritoneal Dialysis (PD) patients includes various potential culprits such as the gradual loss of residual renal function, fluid overload, the endotoxinemia burden, imbalance of adipokines as well as the biocompatibility of the peritoneal dialysis solutions utilized (8, 9). Classical markers of inflammation such as C-reactive protein (CRP) and interleukin-6 levels are frequently increased in PD patients which in turn adversely affects cardiovascular risk as well as technique and patient survival (10, 11). The evaluation and validation of various biomarkers in PD as potential tools for improving patient management is currently a subject of extensive research (12). Accordingly, the associations of immune cell subpopulations as potential markers of inflammations with specific modality related as well as clinical outcomes remain to be determined in PD patients.

Thus, we conducted a pilot study in a cohort of PD patients so as to investigate the profile of specific subpopulations of immune cells in the circulation and their potential correlations with phenotypes of established cardiovascular disease (CVD), including coronary artery disease (CAD) and heart failure (HF), as well as related clinical and laboratory indices. In addition, associations of immune cells with the peritoneal membrane characteristic, dialysis adequacy and various inflammatory and nutritional markers were sought.

## Materials and Methods

### Study Population

**Figure 1:**
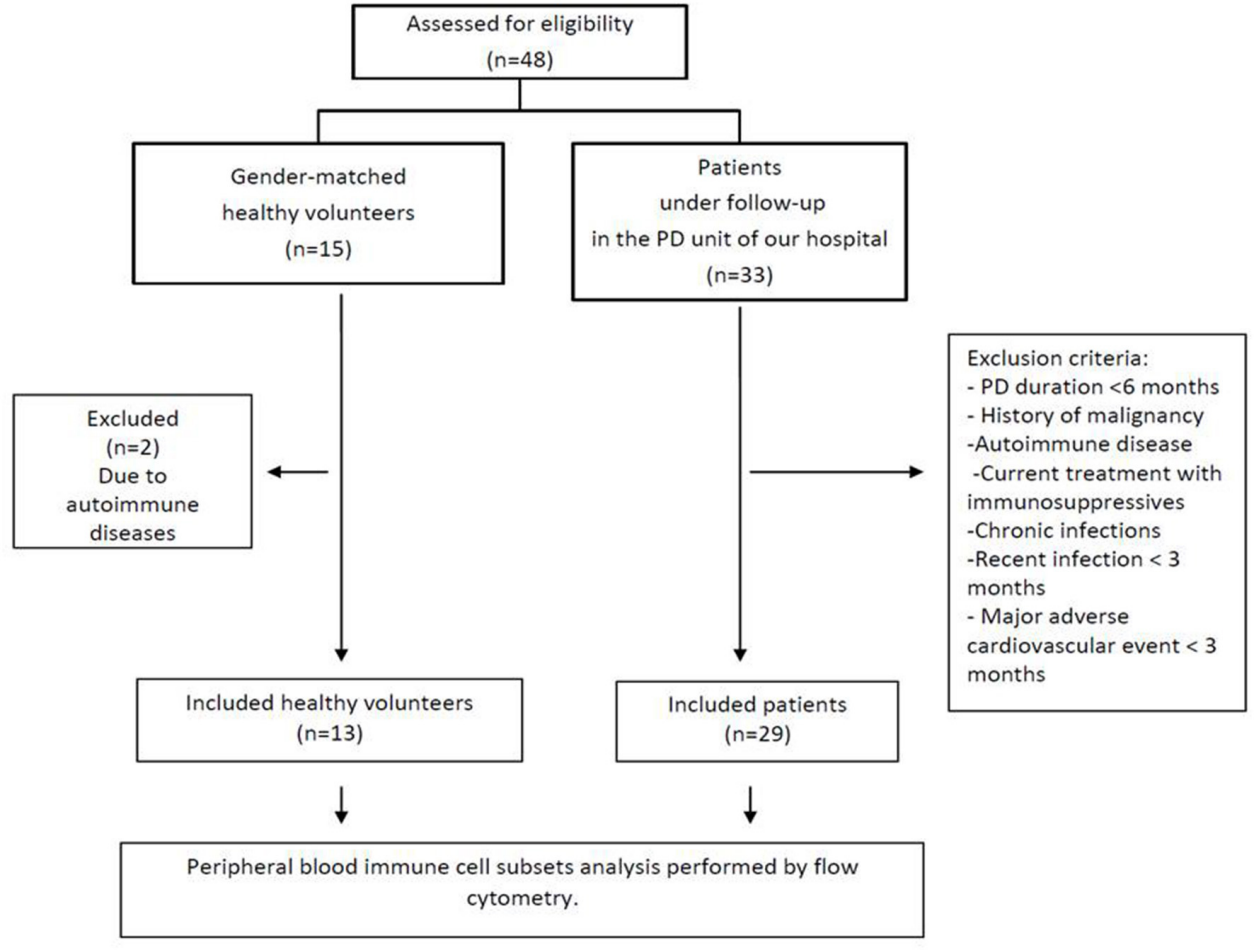
Flowchart of the study.

Twenty-nine stable patients receiving PD for at least 6 months and under follow-up in our PD unit were enrolled in our study together with 13 healthy volunteers so as to compare levels of immune cells in the circulation. Exclusion criteria included a history of malignancy, autoimmune disease, current treatment with immunosuppressive medications and chronic infections. Additionally, patients with a recent (<3 months) infection or major adverse cardiovascular event were excluded from the study (Figure 1). The comorbidities of all the patients including presence of diabetes mellitus (DM), CAD, peripheral artery disease (PAD) and HF were recorded by evaluation of their medical records. All patients provided signed informed consent. The study protocol was approved by the Ethical Committee of our hospital (5/26-3-2020) and has been registered on ClinicalTrials.gov (NCT04286477).

### Laboratory Methods

**Figure 2:**
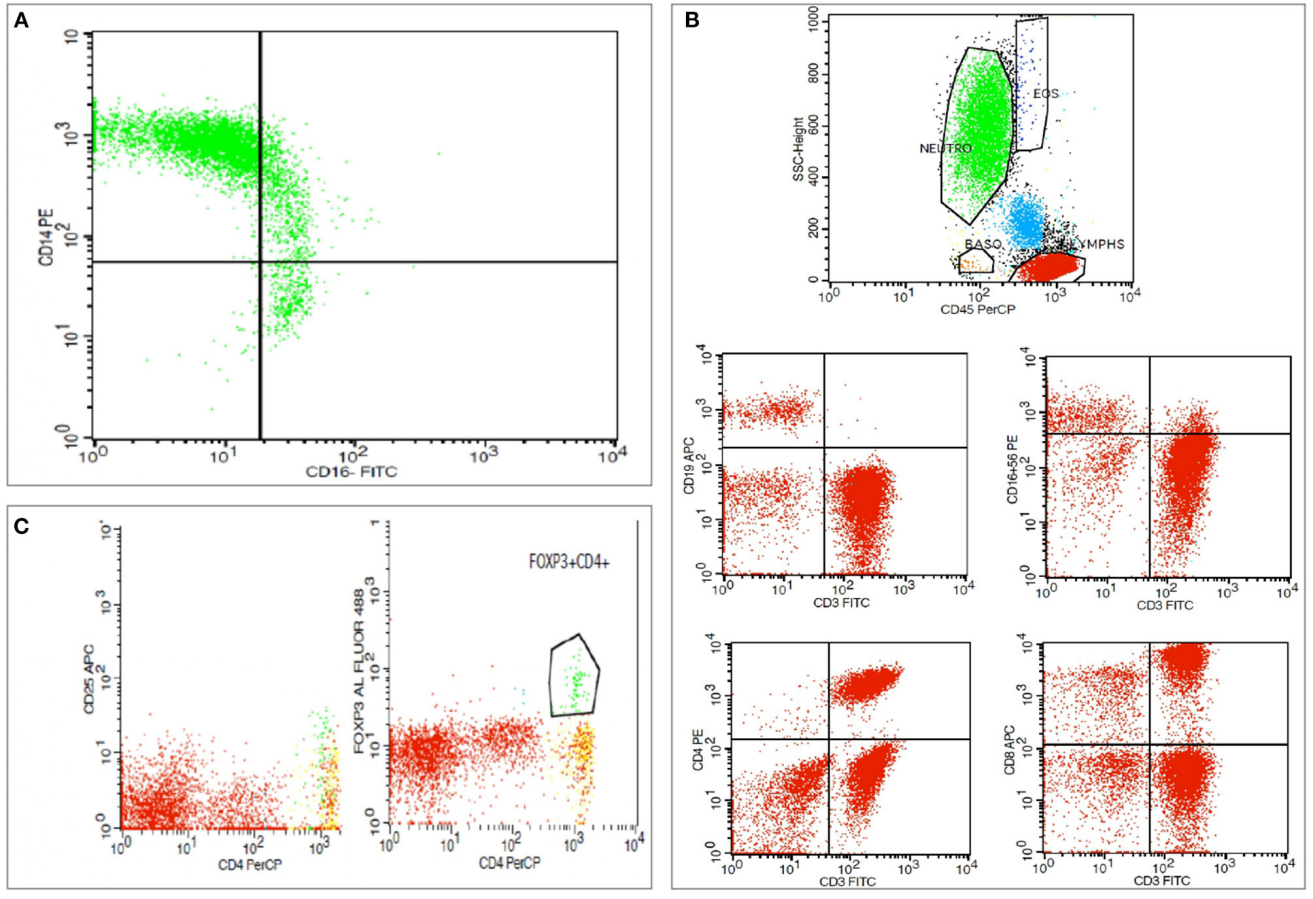
Flow cytometric analysis of a peritoneal dialysis patient. **(A)** Representative dot plots depicting monocyte subsets, differentiated according to their surface expression pattern of CD14 and CD16 in CD14++CD16–, CD14++C16+ and CD14+CD16+ subpopulations. **(B)** Representative dot plots depicting lymphocyte gating with B-lymphocytes, and T lymphocytes, natural killer (NK) cells defined as CD16+CD56+ cells, CD4+ T cells, CD8+ T cells. **(C)** Representative dot plots depicting regulatory T cells (Tregs) defined as CD4+ FoxP3+ CD25^high^ positive cells.

The peripheral blood immune cell subsets analysis was performed by flow cytometry (FC) in a whole-blood assay using 100 μl of whole blood, within 8 h from blood sample withdrawal. Ethylenediaminetetraacetic acid (EDTA) blood-collecting tubes were used for the collection of 2 ml of whole-blood samples from patients. The following monoclonal antibodies were used for analysis: CD45(BD), CD14(BD), CD16(BD), CD4(BD), CD8(BD), CD56(BD), CD3(BD), CD19(BD), CD25(BD), and Fox-P3(eBioscience™). Immune cells subtypes were analyzed using flow cytometry (FACSCalibur) and Cell Quest and FACSDiva software (BD Biosciences). 100 μl of whole-blood was added to flow cytometry tubes and incubated with respective antibodies according to manufacturer's instructions. 500 μl of Versalyse (Beckman Coulter) was added and incubated for 10 min at room temperature (18–25°C) protected from light, to lyse red blood cells. Samples were processed immediately for flow cytometry analysis. The data were analyzed using the CellQuest V3.1 software (Becton Dickinson). Accordingly, CD14++CD16-, CD14++CD16+, and CD16+ percentage and absolute number of cells out of the total monocytes were measured. Additionally, NK cells (CD3+CD16+56+), CD3-CD19+ B lymphocytes, CD3+ CD4+ T cells, CD3+CD8+ T cells, and Tregs (CD4+CD25+ FoxP3+) absolute values and percentage out of the total lymphocytes were measured (Figure 2). Blood was drawn from all subjects under standardized conditions samples were analyzed using standard techniques. Complete blood counts with differential counts of the white blood cell and conventional inflammatory markers including C-reactive protein (CRP), erythrocyte sedimentation rate (ESR) and fibrinogen were measured. Furthermore, serum levels of total protein, albumin, total cholesterol, triglyceride, high-density lipoprotein (HDL) cholesterol, low-density lipoprotein (LDL) cholesterol, calcium, phosphorus, intact parathyroid hormone (iPTH), 25(OH)-vitamin D and ferritin were also determined. High sensitivity troponin I (hsTnI) was measured as a subclinical index of myocardial damage.

All patients underwent bioimpedance analysis of body composition and fluid status using Fresenius Body Composition Monitor (BCM) for determination of overhydration (OH), extracellular water (ECW), intracellular water (ICW), total body water (TBW) content and the OH/ECW index, simultaneously with analysis of immune cell subsets (13). A lung ultrasound examination (Vscan™ GE Healthcare's) was likewise simultaneously performed with estimation of extravascular lung water by counting vertical “comets” or “B-lines and their sum number (14, 15). US-B lines assessment was made in supine position with scanning of the anterior and lateral chest from the second to the fourth intercostal space on the left side and from the second to the fifth intercostal space on the right side, at the parasternal to midaxillary lines as already defined by previous studies (14).

In addition, echocardiographic data from ultrasounds performed within 1 month from immune cell subset analysis were recorded, including left ventricular mass (LVM) and left ventricular mass index (LVMI), left ventricular ejection fraction (EF) and E/E' ratio. Residual renal function (eGFR) defined as the urinary clearance of urea in ml/min and PD adequacy expressed as weekly renal plus peritoneal KT/V of urea, peritoneal transport characteristics as determined by performance of modified PET (16), as well as the normalized protein catabolic rate (nPCR) were recorded.

### Statistical Analysis

Descriptive statistics are reported as means ± standard deviations in normally distributed continuous variables, medians and interquantile range in skewed continuous variables and percentages in dichotomous variables. Normal distribution of all continuous variables was tested with the parametric Shapiro-Wilk normality test. Box cox transformation was applied to transform skewed variables (inverse, or log-transformation). In cases normality was not achieved by any transformation variable was transformed to dichotomous using median as cut-off. Differences between cases and controls were assessed by independent samples *t*-test or non-parametric Mann Whitney test, in normally and skewed continuous variables, respectively. Differences between categorical variables were estimated using 2x2 tables and applying chi-square or fisher's exact test, when applicable.

Univariate analysis was performed for any variable of interest. Any variable having a significant univariate test at a significance level of 0.1 was selected as a candidate for the multivariate analysis in order to identify independent predictors of the dependent variable. In the iterative process of variable selection in multivariate analysis, covariates were removed from the model if they were non-significant or not confounders. Linear regression or logistic regression analysis was used when applicable. Analysis was performed by STATA package, version 14.2 (StataCorp, College Station, TX).

## Results

The main laboratory, echocardiographic and bioimpedance analysis data of the 29 PD patients enrolled are presented in Table 1. The mean age of the study cohort was 64 years ± 14.3 and 58.6% were males. The median dialysis vintage was 34.5 months (IQR 3.2–141). Primary renal diseases included diabetic nephropathy (seven patients, 24.14%), IgA nephropathy (five patients, 17.24%), whereas the cause of nephropathy was unknown in 12 patients (41.38%). Ten patients were diabetics, while CAD was present in seven patients (24%), peripheral artery disease (PAD) in seven patients (24%), with 11 (38%) patients overall displaying atherosclerotic cardiovascular disease (ACVD). In addition, five patients had HF (17.2 %) and echocardiographic evidence of left ventricular hypertrophy (LVH) was present in 20 patients (71.4%).

**Table 1:** Laboratory, lung ultrasound, bioimpedance and echocardiographic data of the PD patients.

Hemoglobin (g/dl)	10.9 (10.2, 11.8)
Cholesterol (mg/dl)	176.4 ± 40.5
TRG (mg/dl)	167 (110, 254)
HDL (mg/dl)	40.5 (34.0, 47.5)
LDL (mg/dl)	94.1 ± 33.2
Albumin(g/dl)	3.5 ± 0.4
Phosphate (mg/dl)	4.3 ± 0.8
iPTH (pg/ml)	235 (127, 306)
vitD (ng/ml)	8.7 ± 2.8
CRP (mg/L)	3 (2, 5)
Fibrinogen (mg/dl)	517 (453, 559)
hsTroponin (ng/ml)	12.6 (6.5, 20.0)
Ferritin(ng/ml)	215 (95, 476)
D/P creatinine	0.73 ± 0.1
KT/V	2.0 ± 0.4
RRF(ml/min)	4.5 (2.3, 6.3)
nPCR (g/kg/day)	0.86 ± 0.16
ECW (L)	15.9 ± 3.3
TBW (L)	33.4 ± 6.6
OH/ECW	0.12 (−0.25, 0.95)
Lung comets ≥ 2	11 (38%)
LVM (g)	218.2 ± 97.8
LVMI (g/m2)	130.5 ± 41.3
E/E'	10.7 ± 4.3
EF %	60 (47.5, 65.0)

*Values are expressed in mean (±SD) or median(IQR 25–75^th^ percentiles). D/P creatinine, dialysate to plasma ratio of creatinine; EF, ejection fraction; ECW, extracellular water; iPTH, intact parathormone; LVM, left ventricular mass; LVMI, left ventricular mass index; nPCR, normalized protein catabolic rate; OH, overhydration; RRF, residual renal function; TBW, total body water*.

### Distribution of the Immune Cell Subpopulations in the PD and Control Groups

Overall, PD patients had 527 ± 199 monocytes and 1731 ± 489 lymphocytes while mean percentage of CD14++CD16+ monocytes was 9.3 ±6.36% (normal range 2–8%), NK cells 16.6 ± 10.3% (normal range 5–15%) and Tregs 2.1 ± 1.76% (normal range 1–3%). Table 2 depicts the measurements results of the immune cell subpopulations in our cohort and the control group.

**Table 2:** Immune cell subpopulations in the control and PD group.

	**Normal controls**	**PD patients**	***P*-value**
WBC	7368.4 ± 1584.4	7451.7 ± 2880.1	0.92
Monocytes	6.4 ± 1.07	6.29 ± 2.02	0.86
CD14++CD16-	88.8 (85.7, 92.5)	87.9 (79.2, 0 90)	0.32
CD14+CD16++	4.11 (2.08, 4.88)	4 (2.5, 6.22)	0.87
CD14++CD16+	4.75 ± 2.75	9.28 ± 6.36	0.002
Lymphocytes (%)	31.3 ± 5.54	22.3 ± 6.28	<0.001
T-lymphocytes (%)	76.09 ± 7.11	76.8 ± 10.1	0.82
B-lymphocytes (%)	9.72 ± 3.63	6.39 ± 3.75	0.01
NK cells (%)	13.2 (8.62, 18.1)	15.4 (9.67, 20.5)	0.54
Tregs (%)	1.85 (1.48, 2.45)	1.79 (1.34, 2.62)	0.71
CD4+ T cells (%)	46.8 ± 7.65	50.1 ± 12.5	0.39
CD8+ T cells (%)	28.9 ± 9.24	25.6 ± 11.2	0.35
CD4CD8 ratio	1.74 (1.19, 2.24)	1.91 (1.40, 3.18)	0.36

*Values are expressed in mean (±SD) or median (IQR 25–75th percentiles). CD, cluster of differentiation; NK, natural killer; Tregs, T regulatory cells*.

**Figure 3:**
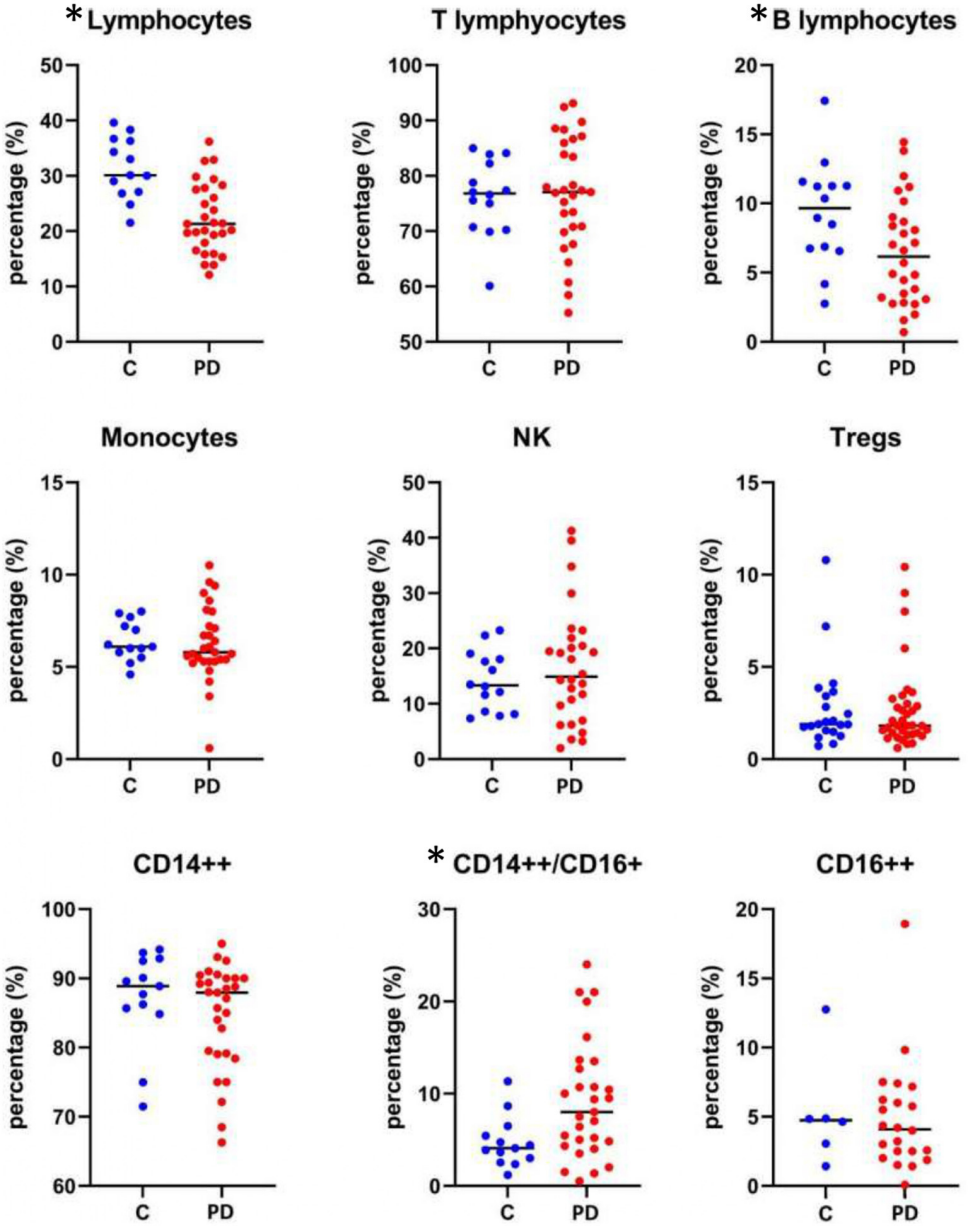
Immune cells subpopulations counts in control and PD patients. *p < 0.05, Values are expressed as mean or medians.

Following comparison of the immune cell subpopulations of the PD patients with the control group, we found that PD patients had lower overall total lymphocytes and B-lymphocytes as well as higher CD14++CD16+ monocytes numbers (Figure 3).

### Correlations of Immune Cell Subpopulations With Clinical Characteristics, Peritoneal Transport Status and Inflammatory Markers of PD Patients

We sought to determine potential correlations between immune cells subpopulations and other clinical characteristics and laboratory parameters in PD patients. Accordingly, patients with higher NK cell levels (>15.4%, *n* = 15) were more likely to be rapid transporters in the modified PET test (D/P creatinine 0.76 ± 0.1 vs. 0.69 ± 0.08, *p* = 0.04). Additionally, patients with higher NK cell levels (>15.4%) had higher cholesterol levels (191.2 ± 47.1mg/dl vs. 160.64 ± 25.1mg/dl, *p* = 0.03) as well as higher CRP levels [2.5 (2, 5) mg/L vs. 5 (3, 9) mg/L, *p* = 0.06]. However, in multiple logistic regression analysis, only the D/P creatinine ratio (odds ratio 7.5; 95% confidence interval, 1.13–50.01; *p* = 0.036) and the total cholesterol levels (odds ratio 1.09; 95% confidence interval (CI), 1.01–1.18; *p* = 0.027) remained significant independent predictors of NK levels.

Regarding Tregs, significant correlations were found between Tregs levels with age and the nPCR, with patients displaying higher percentage of Tregs (>1.79%) being older (70.8 ± 10.7 years vs. 57.7 ± 14.7years, *p* = 0.011) and having a higher nPCR (0.83 ± 0.14 vs. 0.91 ± 0.17, *p* = 0.09).

### Correlations of Immune Cell Subpopulations in PD Patients With Indices of Overhydration and Phenotypes of CVD

With regard to monocytes subtypes, an inverse correlation was detected between CD14++CD16+ % levels and the presence of ACVD (β-coefficient = −5.57, *p* = 0.019). Patients with higher NK cell levels had a higher prevalence of CAD (40 vs. 28.6%, *p* = 0.039) as well as higher E/E'ratios in cardiac ultrasound (12.57 ± 4.34 vs. 8.78 ± 3.53, *p* = 0.02). Patients with higher percentage of Tregs (>1.79%) were more likely to manifest LVH (92.8 vs. 50%, *p* = 0.012), a correlation however which was not maintained following multiple regression analysis.

Regarding phenotypes of CVD, patients with prevalent CAD in comparison to patients without known CAD were diabetics (71.4 vs. 22.7%, *p* = 0.018), had higher CRP, fibrinogen and albumin levels [9 mg/L (5,38), 576 mg/dl (544,737), 3.5 g/dl (3.4,3.7) vs. 3 mg/L (2,4), *p* = 0.0004, 486.5 mg/dl (444,530), *p* = 0.004, 3.1 g/dl (3,3.4), *p* = 0.005], respectively and had a higher E/E' ratio in heart ultrasound (14.08 ± 5.42 vs. 9.65 ± 3.49, *p* = 0.018). Additionally, PD patients with prevalent CAD had NK cells levels elevated above median values (85.7% vs. 40.9%, *p* = 0.04) as well as a lower percentage of B cells (3.85 ± 2.46 vs. 7.2 ± 3.77%, *p* = 0.03). In multiple logistic regression analysis, the percentage of NK levels and of B cells remained an independent significant predictor of the presence of CAD. Thus, patients with increased NK cell levels (>15.4%) had 3.8 times higher risk of CAD comparing with patients with lower NK cell levels (95% CI, 1.86–77.87; *p* = 0.034). On the other hand, the percentage of B cells was inversely associated with the presence of CAD (increase of B-lymphocyte by 1% was independently associated with 30% less risk for presence of CAD (95% CI, −0.71–0.01; *p* = 0.05).

Peritoneal dialysis patients with ACVD as compared to patients without ACVD (*n* = 18) were older (71.2 ± 8.81 years vs. 59.6 ± 15.5, *p* = 0.03) and mainly diabetics (63.6 vs. 16.6%, *p* = 0.01), had lower serum albumin and LDL levels (3.3 ± 2.78 g/dl, 78.8 ± 29.2 mg/dl vs. 3.61 ± 0.37g/dl, *p* = 0.02, 103.7 ± 32.7 md/dl *p* = 0.06, respectively), higher CRP levels [6 mg/L (3,9) vs. 3mg/L (2,4), *p* = 0.01] and displayed higher E/E' ratio in cardiac ultrasound (13.5 ± 5.21 vs. 9.16 ± 2.94, *p* = 0.01). The percentages of CD14++CD16- monocytes and Tregs were significantly higher in patients with ACVD [88.4 ± 8.67 vs. 82.2 ± 8.44, *p* = 0.02 and 1.82 (1.71, 3.45) vs. 1.49 (1.26, 2.08), *p* = 0.01, respectively] while the percentage of CD14++CD16+ monocytes was lower in this patient group [5.2 (2.0, 7.5) vs. 7.49 (4.84, 10.15), *p* = 0.017]. In multiple logistic regression analysis, apart from presence of DM as well as serum albumin and CRP values, the percentages of the CD14++CD16+ monocytes and the Tregs were significantly associated with the presence of ACVD; increase of CD14++CD16+ up to 1% was associated with 31% less risk for ACVD (OR 0.69; 95% CI, 0.48–0.98; *p* = 0.041) and increase of Tregs up to 1% was associated with 20 times higher risk for ACVD (OR 20.5; 95% CI, 1.5–274.7; *p* = 0.022).

Additionally, we examined if patients with evidence of overhydration (defined by the presence of lung comets in lung ultrasound) had different characteristics and expression of immune cell subpopulations as compared with euvolemic patients. Eleven overhydrated patients as defined by presence of ≥2 lung comets showed evidence of overhydration in bioimpedance measurements [ECV/TBW 0.51 (0.49, 0.53) vs. 0.47 (0.43, 0.50), *p* = 0.03)] as well. Patients with clinical evidence of overhydration as compared to euvolaemic patients, had higher CRP, fibrinogen and hsTnI levels [6 mg/L (5,11), 544 mg/dl (511, 721), 17.4 ng/ml (13.1, 42.7) vs. 3 mg/L (2, 4), *p* = 0.004, 486 mg/dl (422, 549), *p* = 0.05, 10.35 ng/ml (4.7, 13.8), *p* = 0.03, respectively] as well as higher E/E'ratios [13 (11.8, 15) vs. 8.4 (7, 11), *p* = 0.02]. Patients with lower CD14++CD16+ % levels had higher OH/ECV values in bioimpedance analysis (β-coefficient = −0.037, *p* = 0.042). In addition, overhydrated patients had lower percentages of lymphocytes (18.3 ± 4.29% vs. 24.7 ± 6.18%, *p* = 0.006) and higher percentages of NK cells [20.5% (14.3, 23.6) vs. 13.21% (6.23, 19.2), *p* = 0.04)]. In multiple logistic regression analysis the CRP [for every increase of 1 mg/dL, there was 1.43 times higher risk for presence of lung comets (OR 1.43; 95% CI, 1.00–2.05; *p* = 0.04)] and the percentage of lymphocytes [a decrease of 1% is associated with 21% less risk for lung comets (OR 0.79; 95% CI, 0.63–0.99; *p* = 0.04)] were independently associated with the presence of lung comets.

## Discussion

There are scarce data in the literature regarding the expression of specific immune cell subtypes, including the CD14++CD16+ proinflammatory monocyte subpopulation, NK cells and Tregs in patients undergoing PD. In addition the potential associations of immune cells with the indices of dialysis adequacy and overhydration as well as the phenotypes of prevalent cardiovascular disease have not been studied in this patient population until now.

The results of our study showed that patients undergoing PD display elevated levels of the pro-inflammatory CD14++CD16+ monocyte subset as compared to normal individuals, indicating the persistence of the inflammatory milieu in this population. Our findings confirm results from previous studies showing that both hemodialysis and PD patients have increased counts of CD14^++^CD16^+^ monocytes compared to individuals without CKD (17). On the other hand, we found an inverse correlation of CD14++CD16+ levels with presence of ACVD, although longitudinal epidemiological studies have confirmed at large a direct relationship between increased CD14++CD16+ monocytes and occurrence of adverse cardiovascular outcomes in patients with CKD and dialysis patients (18, 19). However, it should be noted that the number of peritoneal dialysis patients evaluated by these studies was very small, with only one study including <20 peritoneal dialysis patients (17). Our finding support a suggested J-shaped relationship that might exist between CD16+ monocyte subsets and adverse outcomes in patients receiving hemodialysis, such that both high and low CD16+ counts confer an increased risk of all-cause and cardiovascular mortality (20). Moreover, PD patients with ACVD were found to have higher levels of classical CD14++CD16-monocytes. It should be noted that available data in the literature remain controversial with regard to the specific status and role of the classical monocytes in patients with ACVD with or without CKD (20–23).

The multifaceted nature of NK cells and their role in the propagation vs. modulation of inflammation remains a subject of dispute. In addition, it should be noted that inflammation itself has been associated both with induction of NK cell apoptosis and augmented proliferation in the setting of cytokine stimulation. Increased NK cell levels in the circulation have been associated with disease activity or adverse prognosis in several disease models of inflammation, such as sepsis and autoimmune disease (24–27). Accordingly, both in experimental sepsis models and in clinical studies of patients with sepsis and septic shock, NK cells in the circulation increased in numbers and displayed an activated phenotype whereas their counts showed a direct association with mortality (24–26).

Furthermore, faster peritoneal transport status in PD patients has been associated among others with intraperitoneal and systemic inflammation. We have found a direct correlation between increased NK cell counts and fast peritoneal transport status in our cohort (28). Moreover, available data suggest that fluid overload is significantly and reciprocally associated with systemic microinflammation and it is more frequent in fast trasporters (29). Our study results indicate that increased NK cells were linked to fluid overload in PD patients, determined either as overhydration in lung ultrasound, BCM measurements or as an increased E/E' in heart ultrasound.

Although NK cells are suspected to play a direct role in atherogenesis considering their abundance in the necrotic cores of atherosclerotic plaques, it remains controversial whether they are harmful or protective toward the vascular tissues (30). Experimental models have shown that depletion of functional NK cells decreases the atherosclerosis burden in atherosclerosis- susceptible LDL receptor null mice (31). On the other hand, a recent study in mice, lacking or having hyper-responsive NK cells, showed that the atherosclerotic burden in the aortic sinus and in the descending aorta did not change, thus suggesting that these cells have no effect on the pathogenesis of atherosclerosis (32). In our study, increased NK cell levels in PD patients were associated with increased risk for prevalent CAD. Although some clinical studies have found reduced NK cell counts and cytotoxic activity in patients with prevalent CAD, others have shown not only increased levels of total circulating NK cells in atherosclerotic patients but a direct relationship between NK cell counts and complications in these patients as well (6, 33–35).

Finally, we did not detect any significant differences in NK cell counts between PD patients and healthy subjects. Previous studies have yielded controversial results regarding NK cell counts in patients undergoing hemodialysis or peritoneal dialysis (36–38). However, it has been suggested that lower NK cell counts directly correlate with the glomerular filtration rate (GFR) in hemodialysis patients, thus allowing us to speculate that the preservation of residual renal function as occurs in PD, might have affected our results (36).

Our results confirm results of earlier studies regarding total lymphocytes and B-lymphocyte depletion due to an increased apoptosis in patients with ESKD undergoing dialysis (39, 40). In addition, we found an inverse association of the total lymphocytes count and percentage of B cells with overhydration and the presence of CAD respectively in PD patients. Reduced total lymphocyte count is an established independent predictor of mortality in heart failure patients whereas with regard to atherosclerosis, the mode that B lymphocytes affect the atherosclerotic lesions currently remains a subject of ongoing investigation (41, 42). Likewise, CD19^+^ B-cell lymphopenia has been suggested as an independent predictor of all-cause and CV mortality in hemodialysis patients (40).

With regard to Tregs, the influence of dialysis on their counts and function remains to be further clarified (43–45). Thus, a recent meta-analysis showed that ESKD patients not undergoing dialysis displayed a lower percentage of Tregs on CD4+ T-cells compared to healthy individuals, but on the other hand no significant difference was observed with respect to Tregs percentage between hemodialysis patients and healthy individuals (44). We found no significant differences between the percentage of Tregs on total lymphocytes and normal controls. A great deal of experimental and clinical evidence indicates a beneficial cardioprotective role of Tregs, associating their reduced numbers and impaired function with various models of cardiovascular diseases, including atherosclerosis, hypertension and heart failure (46). On the other hand, we found that patients with increased percentage of Tregs were more likely to be older and have LVH or ACVD. Whether this finding should be ascribed to a compensatory mechanism or specific immunologic properties of the Tregs themselves remains to be elucidated by future studies. Similarly, a study investigating whether the levels of circulating Treg cells relate to the degree of atherosclerosis showed an increase in Tregs only in patients with acute coronary syndromes, whereas patients with stable angina Tregs we not altered compared to healthy control subjects (47). In addition, no difference in regulatory T cells was observed between type 2 diabetes mellitus patients with cardiovascular disease as compared to those without (48).

To our knowledge, this is the first study to evaluate the association of the profiles of immune cells subpopulations with peritoneal transport characteristics, indices of overhydration and phenotypes of cardiovascular disease in a cohort of long-term PD patients. Yet, there are limitations to our study, including a relatively small sample size as well as its observational and cross-sectional nature. Moreover, a relatively small number of the patients included had prevalent CAD or ACVD and overt overhydration. Finally, only the phenotypes of immune cell subpopulations were studied but not their function or association with other immune markers, which is the aim of another study that our group is currently conducting.

The state of pro-inflammation and immune deregulation appear to persist after initiating PD. Future research is required to evaluate the role of immune cells subsets as potential tools to identify patients who are at the highest risk for complications and to guide interventions that may improve clinical outcomes.

## Data Availability Statement

The raw data supporting the conclusions of this article will be made available by the authors, without undue reservation.

## Ethics Statement

The studies involving human participants were reviewed and approved by University Hospital of Ioannina Ethics Committee. The patients/participants provided their written informed consent to participate in this study.

## Author Contributions

All the authors have substantially contributed to the conception, design, and research conduct as well as to the writing of the manuscript. All authors contributed to the article and approved the submitted version.

## Conflict of Interest

The authors declare that the research was conducted in the absence of any commercial or financial relationships that could be construed as a potential conflict of interest.

## Publisher's Note

All claims expressed in this article are solely those of the authors and do not necessarily represent those of their affiliated organizations, or those of the publisher, the editors and the reviewers. Any product that may be evaluated in this article, or claim that may be made by its manufacturer, is not guaranteed or endorsed by the publisher.
